# Immunohistochemical based molecular subtypes of muscle-invasive bladder cancer: association with HER2 and EGFR alterations, neoadjuvant chemotherapy response and survival

**DOI:** 10.1186/s13000-023-01295-y

**Published:** 2023-02-03

**Authors:** Duaa S. Helal, Sara A. Darwish, Radwa A. Awad, Dina A. Ali, Dina M. El-Guindy

**Affiliations:** 1 Pathology Department, Faculty of Medicine, Tanta University, Tanta, 31527 Egypt; 2 Clinical Oncology and Nuclear Medicine Department, Faculty of Medicine, Tanta University, Tanta, Egypt; 3 Clinical Pathology Department, Faculty of Medicine, Tanta University, Tanta, Egypt

**Keywords:** Muscle invasive bladder cancer, Molecular classification, GATA3,CK5/6,p53,HER2,EGFR, Luminal, Basal, Response to chemotherapy, Survival

## Abstract

Muscle-invasive bladder cancers (MIBCs) is a group of molecularly heterogonous diseases that could be stratified into subtypes with distinct clinical courses and sensitivities to chemotherapy. Clinical application of molecular subtypes could help in prediction of neoadjuvant chemotherapy (NAC) responders. Immunohistochemical (IHC) markers such as GATA3, cytokeratin (CK) 5/6, and p53 are associated with these subtypes and are widely available. Human epidermal growth factor receptor 2 (HER2) and epidermal growth factor receptor (EGFR) are mutated in multiple cancers including MIBC and are potential therapeutic targets. HER2/EGFR status of MIBC subtypes has not been investigated. Tissue microarrays (TMAs) were constructed from transurethral resection of the bladder tumor (TURB) specimens and stained with GATA3,CK5/6,p53 and HER2 in addition to Quantitative Reverse Transcription PCR for detection of EGFR gene. Of the total cases, 45% were luminal, 36.7% basal and 18.3% p53 wild subtype (p53-WT). Univariate analysis showed that overall survival (OS) and disease-free progression survival (DFS) were significantly longer for luminal subtype. In multivariate analysis, molecular subtype, HER2 status and LV invasion were independent prognostic factors for DFS and OS. Basal subtype showed a significantly better response to NAC. HER2 expression was significantly higher in luminal while EGFR expression was significantly higher in basal subtype. Kaplan-Meier survival curves revealed a significant longer OS and DFS for HER2 negative than positive cases. MIBC can be stratified using a simple IHC panel [GATA3,CK5/6,P53] into clinically relevant prognostic molecular subtypes. Basal tumors are aggressive and respond well to NAC while luminal have better OS. P53-WT tumors are chemoresistant and require further treatments. HER2 and EGFR are potential therapeutic targets for molecular subtypes of MIBC where luminal tumors are more likely to benefit from HER2 and basal from EGFR directed therapies.

## Introduction

Muscle invasive bladder carcinomas (MIBCs) are highly heterogeneous tumors. Identifying patients who have a higher possibility of response to neoadjuvant chemotherapy (NAC) using molecular subtype classification appears to be a promising strategy to improve survival and prevent undesirable toxicity. Gene expression profiles of basal and luminal subtypes of urothelial carcinoma are mostly reflective of the normal expression signatures of basal and intermediate/luminal layers of the normal urothelium, respectively [1].

Molecular profiling of specimens from large cohorts of bladder carcinoma have prompted their classification into molecular subtypes that show specific genomic and transcriptomic features, like those reported in breast cancer. These subtypes may exhibit distinct associations with treatment response and patient survival [2, 3].

MIBCs were divided into two subtypes in a study by the University of North Carolina. P53-like tumors were added to the MD Anderson classification (MDA). Up till now, several other molecular classifications have been proposed, some of which overlap. When compared to grade and stage, tumor subtyping may provide a more informative description of the tumor biology, which could enhance risk stratification and clinical decision-making [4].

In clinical practice, using whole transcriptome profiling for all patients could be costly and time-consuming. Subtyping of bladder tumors using a small number of immune markers is regarded as an alternative for use in clinical settings because immunohistochemistry (IHC) is an inexpensive, readily accessible, and reliable technique. In spite of the significant overlap between subtypes found in several studies, a small panel of IHC markers, including CK5/6 and CK14 for basal and GATA binding protein 3 (GATA3) and cytokeratin 20 (CK20) for luminal tumors, revealed significant differential expression patterns [5].

Recently, genetic elucidation of carcinogenesis and developing targeted cancer therapies have rapidly progressed. In breast and gastric cancers, Erb-b2 receptor tyrosine kinase 2 (ERBB2) known as Human epidermal growth factor receptor 2 (HER2) protein overexpression and/or gene amplification is detected in some cases, and significant prognostic improvement has been achieved using therapeutic drugs targeting HER2 (eg, trastuzumab) [6]. HER2 antibodies are widely used to lower the incidence of tumor recurrence and cancer-related mortality in breast carcinoma [7]. Recent studies have reported a similar HER2 protein alteration in a subset of urothelial carcinomas, but its clinical implication has not been investigated. Moreover, despite several trials studying HER2 as a target in bladder carcinoma, no general conclusions have been reached [8].

The epidermal growth factor receptor (EGFR) is a member of the ErbB family of receptor tyrosine kinases and performs important functions in physiology of epithelial cells. It is mutated and/or overexpressed in various cancers including bladder cancers and is the target of multiple therapies currently used in the clinical practice such as lung carcinoma. Anti-EGFR therapy combined with traditional treatment may improve the prognosis for MIBC [9]. Disparate clinical results for EGFR and HER2 targeted therapies in MIBC is most likely explained by the underlying heterogeneity of bladder cancer and the lack of adequate molecular descriptions of its molecular subtypes; the context in which the HER2 and EGFR targets operate [10].

The aim of the present work was to identify the molecular subtype of MIBC cases using the immunohistochemical markers; GATA3, CK5/6 and p53 and study their prognostic significance in relation to clinicopathologic parameters,survival and response to NAC treatment. This study was extended to investigate the expression of HER2 and EGFR in molecular subtypes of MIBC.

## Patient and method

### Study design and patient selection

This prospective study was conducted between June 2017 and December 2021 and included 60 patients diagnosed with MIBC in Pathology, Clinical pathology and Clinical Oncology and Nuclear Medicine Departments, Faculty of Medicine, Tanta University, Egypt. A written informed consent had been collected from all patients included in the study. The study was approved by Research Ethics Committee in Tanta Faculty of Medicine (Code#34536).

#### Inclusion criteria

Patients diagnosed with MIBC after performing transurethral resection of the bladder tumor (TURB), with good performance status.

#### Exclusion criteria

Patient with superficial or metastatic bladder carcinoma or poor performance status were excluded from the study.

#### Diagnostic work up

Careful history was obtained followed by general and local abdominopelvic examination and investigations; laboratory investigations (eGFR is required for cisplatin regimens), abdomino-pelvic ultrasonography, Computed Tomography (CT) scan abdomen and pelvis with oral and intravenous contrast and T and N were assessed accordingly. Chest CT scan and bone scan were performed if needed**.**

#### Neoadjuvant chemotherapy (NAC)

Chemotherapy regimens received were cisplatin 70 mg/^m2^ IV on day 1 and Gemcitabine 1000 mg/^m2^ IV on days 1 & 8 (the cycle was repeated every 21 days for 4 cycles). Patients who received Carboplatin the dose was Area Under the Curve (AUC) 4 to 5 IV on day 1 (the cycle was repeated every 21 days for 4 cycles) followed by cystectomy.

#### Adjuvant treatment

Adjuvant radiotherapy was received in patients with T3, T4, positive margin or positive lymph nodes in non-responders.

#### Survival and follow-up

Patients we routinely evaluated every 3 months during the first year and after that every 6 months.

#### Outcome measures

The main outcome data during the follow up period included disease-free survival (DFS) and overall survival (OS). DFS was identified as the time from date of diagnosis to clinical or radiological evidence of disease progression. OS was identified as the time from date of diagnosis to date of death, or date of last follow-up. Complete response (CR) to NAC was defined as no residual tumor by CT scan and no malignant cells in cystectomy specimens. Cases were classified as responders if CR was achieved. If CR was not achieved; cases were classified as non-responders [11]. The primary end point of the study was DFS while the secondary end point was OS.

### Histopathological evaluation

All preoperative TURB and post NAC cystectomy specimens were subjected hematoxylin and eosin (H&E) staining and examination to confirm the diagnosis of MIBC. Tumors were graded according to the World Health Organization grading system [12] and staged according to the TNM staging system [13]. Other data as histological type and presence of lymphovascular (LV) invasion were recorded for each case. Post NACT response was evaluated in cystectomy specimens and pathological complete response was defined as no residual invasive or in situ carcinoma on histological examination [14].

#### Tissue microarray (TMA)

H&E stained slides from TURB specimens of the included cases were scanned to identify well preserved tumor areas. Two tissue cores from selected areas on paraffin blocks were then injected into the holes on the recipient blocks to form TMA Paraffin blocks (6 × 4 array) using the TMA builder mold (CAT# TMA-001, Thermo Fisher Scientific, Runcorn, UK).

### Immunohistochemical staining

Five mm sections were obtained from TMA blocks on positively charged slides and were dried for 30 min at 37 °C. The slides were placed in a Dako PT Link unit for deparaffinization and antigen retrieval. High pH EnVision™ FLEX Target Retrieval Solutions was applied, reaching 97 °C for 20 min. Dako Autostainer Link 48 was used for immunohistochemistry. Slides were placed in Peroxidase-Blocking Reagent for 10 min, incubated with 4 different antibodies GATA3 (BioCare,cat#CN405,1:200,USA), CK5/6 (Biocare,cat#CM105,1:200,USA), p53 (BioCare, cat# CM 042,1:100,USA), Anti-ErbB2 / HER2 (Biocare,cat#ACA 342,1:50,USA) with appropriate positive and negative controls. Afterwards, slides were incubated with horseradish peroxidase polymer reagent for 20 min and diaminobenzidine chromogen for 10 min. Slides were then counterstained with hematoxylin.

### Scoring of Immunohistochemical markers

GATA3 nuclear positivity was assessed with a semiquantitative immuno score, as defined by Remmele and Stegner, multiplying staining intensity score of positive cells (0 = negative, 1 = weak, 2 = moderate, 3 = strong) with the percentage of positive cells (0 = 0, 1 < 10%, 2 = 10–50%, 3 = 51–80%, 4 > 80%). Cancer tissue is considered positive when the Remmele Score is 3–12 [15], For CK5/6, percentage of cells with positive cytoplasmic staining was recorded, and specimens were considered positive if > 50% of cells showed CK expression [16]. Nuclear p53 was scored as 0 if negative, + 1 positive in < 50% scattered tumor cells and + 2 in diffuse > 50% positive tumor cells [17]. HER-2 expression was scored (0, + 1, + 2, + 3) using the College of American Pathologists (CAP) guidelines, where HER2-positive tissue samples show strong and homogenous membranous expression in > 10% of tumor cells; otherwise, they were considered negative for HER2 [18]. Fluorescence in situ hybridization was performed to search for gene amplifications for cases with + 2 score.

### Identification of the molecular subtype

Cases were classified according to the immunostaining profile into luminal, basal or p53-wild subtype following the Choi et al. stratification strategy [19]. Tumors positive for CK5/6 and negative for GATA3 were considered basal; tumors negative for CK5/6 and positive for GATA3 were considered luminal [20]. P53 subtype of cases was assigned according to immunostaining pattern for p53 antibody. P53 mutated phenotype was based on increased diffuse expression (+ 2) or complete absence (0) of nuclear p53 staining in the tumor cells. P53 wild phenotype was determined by nuclear expression in scattered tumor cells (+ 1) and these cases were assigned as p53-WT subtype regardless of their GATA3 and CK5/6 staining result [17].

### Detection of EGFR gene expression by quantitative reverse transcription PCR

TURB samples were taken from the tumor mass and were directly stored at − 80 °C for molecular study. The RNA was extracted from each stored frozen bladder tissue using an RNA extraction kit (RNeasy mini kit, Qiagen, Hilden, Germany, Catalog no. 74104) according to the manufacturer’s protocol. RNA yields were assayed quantitatively by measuring the absorbance at 260 nm on a Jenway UV/Visible Spectrophotometer 6305, Staffordshire, UK. RNAs were reversely transcribed into cDNA using the QuantiTect® Reverse Transcription kit (Qiagen, Hilden, Germany, Catalog no. 205311), according to the manufacturer’s instructions. RT-PCR amplifications with relative quantitation of EGFR mRNA expression were performed using QuantiTect SYBR Green PCR Master Mix kit (QIAGEN, Texas, USA, CAT # 204141). The primers’ sequence for EGFR was as follows: forward 5′-AGGCACGAGTAACAAGCTCAC-3′ and reverse 5′- ATGAGGACATAACCAGCCACC-3′ and for GAPDH as follows: forward primer:5′-ACCACAGTCCATGCCATCCAC-3′;reverse primer: 5′-TCCACCACCCTGTTGCTGTA-3′. The plate was applied on a real-time PCR system (Applied Biosystems, step I version) with the following thermal profile: Hold at 95 °C for 10 seconds, then 40 cycles (denaturation at 95 °C for 15 seconds and annealing/extension at 59 °C for 60 seconds). The cycle threshold (Ct) values were determined for EGFR and GAPDH genes, and relative EGFR gene expression was determined using 2^−ΔCT^ method [21]. Cases were classified into high and low EGFR groups considering the median value as cut off point.

### Statistical analysis of the collected data

Data were handled using Statistical Package for Social Science (SPSS) version 26 (SPSS Inc. Released 2019. IBM SPSS statistics for windows, Armnok, NY: IBM Corp.). Chi-square test (χ2), with Z test to compare column proportions, was used to study association between qualitative variables. Whenever any of the expected cells were less than five, Fischer’s Exact test and Monte Carlo tests were used. Analysis of variances (ANOVA), with homogeneity testing, was used for comparison of quantitative variables between more than two groups of normally distributed data with LSD test as post-hoc test while; Kruskal Wallis test was used for comparison of quantitative variables between more than two groups of not normally distributed data with Tamhane’s test as post-hoc test. OS and DFS were done using Kapaln-Meier statistics with log rank test to express the significance. Cox regression was used for multivariate survival regression analysis. Two-sided *P*- value of < 0.05 was considered statistically significant.

## Results

The study included 60 cases of MIBC. The clinicopathological characteristics of studied cases are summarized in Table 1. The median age of the studied cases was 61.5 years ranging from 45 to 74 years with a strong male predominance (44/60;73.3%). Most of the studied cases were stage T3 (*n* = 25;41.7%), N2 (*n* = 24;39.3%) and all cases were of high histological grade. Conventional urothelial histology was found in 35 cases (58.3%). Twenty-two cases showed areas of squamous differentiation (36.7%) and glandular differentiation was found in only 3 cases(5%). LV invasion was detected in 21 cases(35%). All included cases received neoadjuvant chemotherapy while only 44 cases (73.3%) received adjuvant radiotherapy. Median follow up period for the included cases was 41 months (18–54).

**Table 1:** Clinicopathologic characteristics of the studied cases of MIBC

Clinicopathological variable	Number (%)
Age (years) Median (range)	61.50 (45–74)
Sex	
Male	44 (73.3)
Female	16 (26.7)
Tumor stage	
T2	15 (25.0)
T3	25 (41.7)
T4	20 (33.3)
Histological type	
Conventional urothelial	35 (58.3)
Urothelial with Squamous differentiation	22 (36.7)
Urothelial with glandular differentiation	3 (5.0)
Lymph node extension	
N0	13 (21.7)
N1	23(38.3)
N2	24 (40.0)
Lymphovascular invasion	
Absent	39 (65)
Present	21 (35)

### GATA3, CK5/6 and P53 immunostaining results

**Fig. 1:**
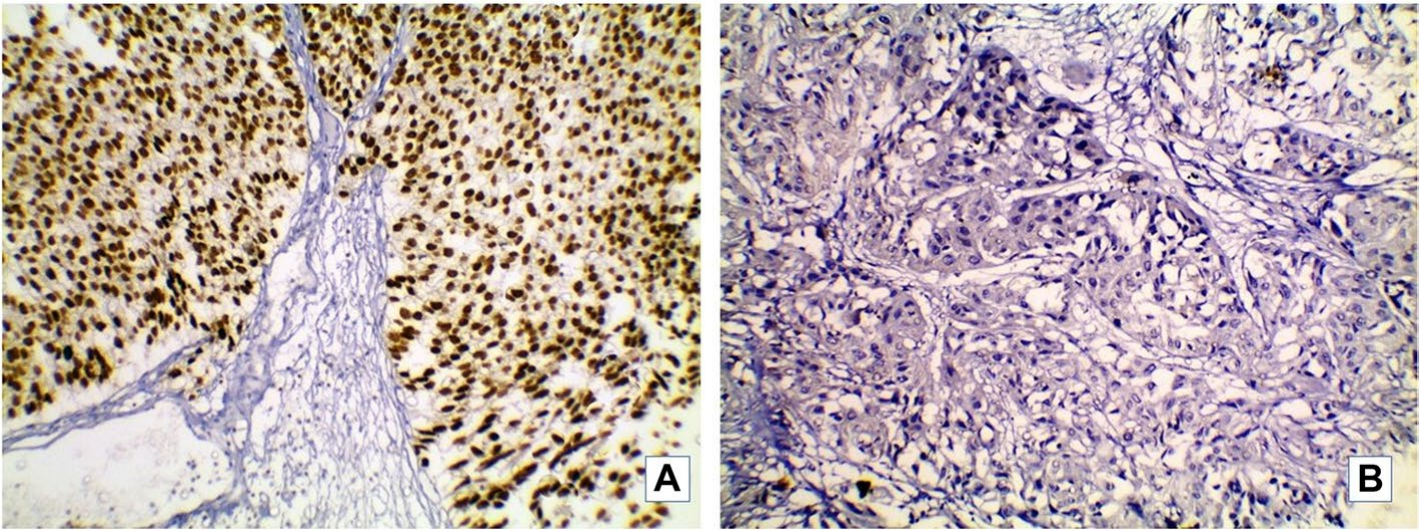
Representative image of GATA3 immunostain in studied MIBC cases. **A**: A case of urothelial carcinoma showing positive nuclear GATA3 (× 200). **B**: High grade urothelial carcinoma with squamous differentiation negative for GATA3 (× 200)

**Fig. 2:**
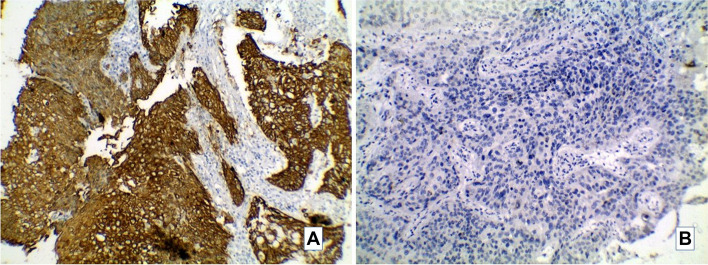
Representative image of CK5/6 immunostain in studied MIBC cases. **A**: A case of UC with squamous differentiation showing positive cytoplasmic CK5/6 (× 200). **B**: Conventional UC negative for CK5/6 (× 200)

**Fig. 3:**
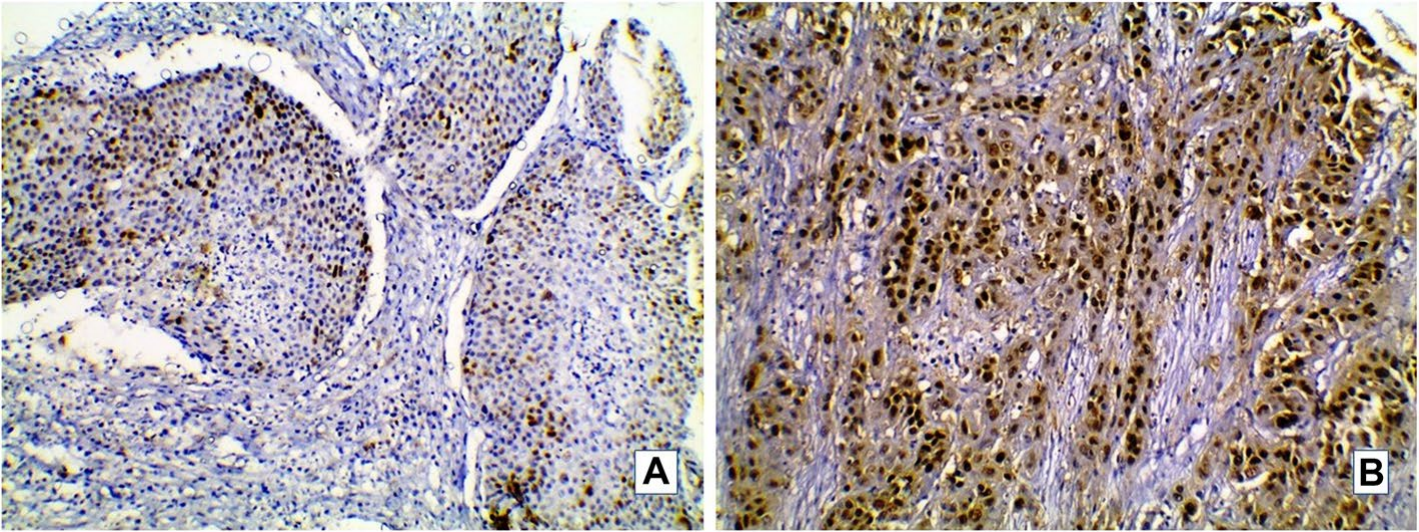
P53 immunostain in studied MIBC cases. **A**: Urothelial carcinoma showing p53 wild type staining score (+ 1) (× 200). **B**: High grade urothelial carcinoma with squamous differentiation showing mutant p53 staining pattern score + 2 (× 200)

GATA3 positivity was detected as brownish nuclear staining of tumor cells. Thirty-six cases (60%) were positive for GATA3 (Fig. 1). The remaining 24 cases(40%) scored from 0 to 2 and were considered negative for GATA3. CK5/6 showed positive brownish cytoplasmic staining in 22 cases (36.7%) (Fig. 2). P53 nuclear staining was positive in 34 cases; eleven cases showed wild type (+ 1) expression (Fig. 3).

### Identification of the molecular subtype

**Fig. 4:**
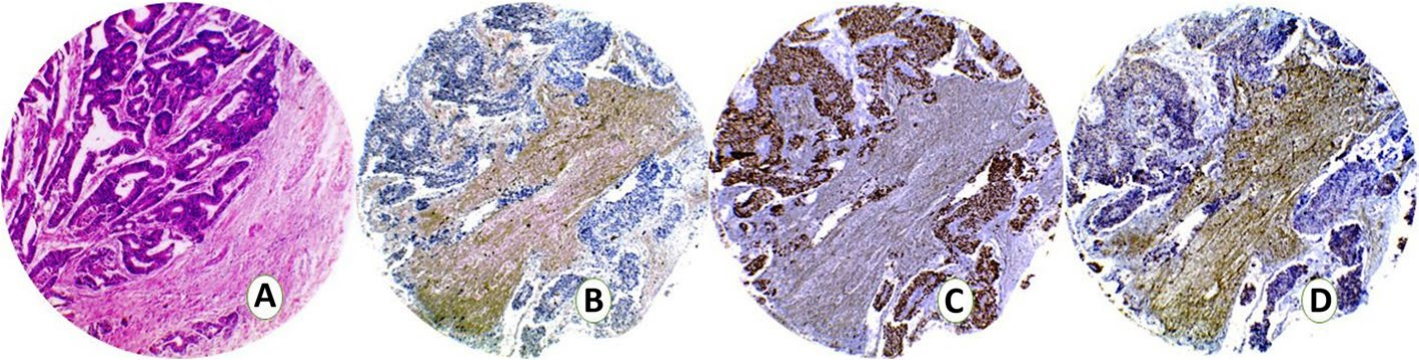
TMA sections from a UC case assigned as luminal subtype showing. **A**: H&E **B**:CK5/6 negative. **C**: GATA3 nuclear positivity. **D**: P53 negative

**Fig. 5:**
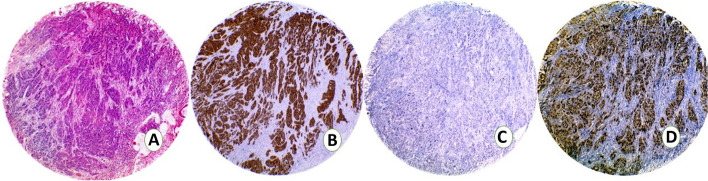
TMA sections from a UC case assigned as basal subtype showing. **A**: H&E **B**:CK5/6 positive. **C**: GATA3 negative. **D**: Diffuse nuclear p53 expression

Cases were categorized as either luminal, basal, or p53-wild subtype according to the immunostaining scores (Table 2). Twenty-seven cases (45%) were classified as luminal tumors as they expressed GATA3 with median Remmele score 11 (range 4–12) (Fig. 4). Twenty-two cases (36.7%) expressed the basal marker CK5/6 and were classified as basal subtype (Fig. 5). Eleven cases (18.3%) showed score + 1 for p53 and were classified as p53-WT.

**Table 2:** Immunoscores of luminal, basal and p53 markers in MIBC

	Luminal No. %	Basal No. %	P53-WT No. %	Total
GATA3				
0–2 [Negative]	0 (0.0)	22 (100.0)	2 (18.2)	24
3–12 [Positive]	27 (100.0)	0 (0.0)	9 (81.8)	36
CK5/6				
Positive	0 (0.0)	22 (100.0)	0 (0.0)	22
Negative	27 (100.0)	0 (0.0)	11 (100.0)	38
P53				
0	7 (25.9)	19 (86.4)	0 (0.0)	26
+ 1 (wild type)	0 (0.0)	0 (0.0)	11 (100.0)	11
+ 2	20 (74.1)	3 (13.6)	0 (0.00)	23

### Clinicopathological characters in MIBC molecular subtypes (Table 3)

There were statistically significant differences in age and sex of cases among different molecular subtypes. Basal subtype showed a significantly younger age (51.82 ± 5.7 years) and female predominance (10/22;45.5%) [*p* values < 0.001 and 0.04 respectively]. Histologic types of tumors were significantly distributed among the molecular subtypes; all basal tumors showed squamous differentiation. Most of luminal tumors (24/27;88.9%) were of conventional urothelial morphology while the remaining 3 cases (11.1%) had a glandular component. Regarding tumor stage, the basal subtype had a significantly higher cases of T4 (13/22;59.1%) than luminal and p53-WT [*p* value =0.021]. Luminal subtype showed significantly fewer LV invasion (5/27;18.5%) than both p53-WT and basal subtypes [*p* value =0.047].

**Table 3:** Relation between molecular subtype and clinicopathologic characteristics of MIBC studied cases

	Luminal *N* = 27; 45%	Basal *N* = 22;36.7%	P53-WT *N* = 11; 18.3%	*P* value
Age (mean ± SD) years	62.81 ± 7.6	51.82 ± 5.7	63.45 ± 7.3	*P* < 0.001*
Sex				*P* = 0.04*
Male	23(85.2)	12(54.5)	9 (81.8)	
Female	4(14.8)	10(45.5)	2(18.2)	
Tumor Stage				*P* = 0.016*
T2	10(37.0)	3(13.6)	2(18.2)	
T3	12(44.4)	6(27.3)	7(63.6)	
T4	5(18.5)	13(59.1)	2(18.2)	
Histologic type				*P* < 0.001*
Conventional Urothelial	24 (88.9)	0 (0.0)	11 (100)	
UC with squamous differentiation	0 (0.0)	22 (100)	0 (0.0)	
UC with glandular differentiation	3 (11.1)	0 (0.0)	0 (0.0)	
Lymph node extension				*P* = 0.002*
N0	8 (29.6)	2 (9.1)	3 (27.3)	
N1	15 (55.6)	4 (18.2)	4 (36.4)	
N2	4 (14.8)	16 (72.7)	4 (36.4)	
Lymphovascular invasion				*P* < 0.001*
Present	5 (18.5)	10(45.5)	6 (54.5)	
Absent	22 (81.5)	12 (54.5)	5 (45.5)	
HER2				*P* < 0.001*
Negative	18 (66.7)	19(86.4)	11(100)	
Positive	9(33.3)	3(13.6)	0(0.0)	
EGFR				*P* < 0.001*
Low	21 (77.8)	0 (0)	9(81.8)	
High	6 (22.2)	22 (100.0)	2(18.2)	
Response to treatment				0.045*
Non responders [non-CR^#^]	22(81.5)	12(54.5)	10(90.9)	
Responders [CR^#^]	5(18.5)	10(45.5)	1(9.1)	
Recurrence				0.127
Positive	6 (22.2)	9 (40.9)	6 (54.5)	
Negative	21 (77.8)	13 (59.1)	5 (45.5)	
Death				0.226
Positive	3(11.1)	4(18.2)	4(36.4)	
Negative	24 (88.9)	18 (81.8)	7 (63.6)	

*P value less than 0.5 was considered statistically significant

# CR: Complete response

### Relation between molecular subtypes of MIBC and survival (Fig. 6A)

Analysis of Kaplan-Meier survival curve revealed that the mean OS was 49.92 months (95% CI, 47.81–52.04). OS was significantly longer for luminal subtype (mean 51.97 months 95% CI: 49.89–54.05) than basal (mean 47.57 months 95% CI: 44.60–50.54) and p53-WT (mean 42.22 months 95% CI:37.96–46.49) [*p* value = 0.047]. The mean DFS of the included cases was 45.00 months (95% CI, 42.47–48.32). The luminal subtype had a significantly longer DFS (mean 49.65 months 95% CI:46.77–52.53) than both basal (mean 43.33 months 95%CI:39.74–46.92) and p53-WT (mean 34.27 months 95% CI:26.64–41.90) subtypes [p value = 0.007]. A multivariate analysis was done to identify the independent prognostic factors of DFS and OS. It was found that molecular subtype, HER2 status and LV invasion were independent prognostic factors for DFS and OS. The details of the multivariate analysis are provided in Table 4.

**Fig. 6:**
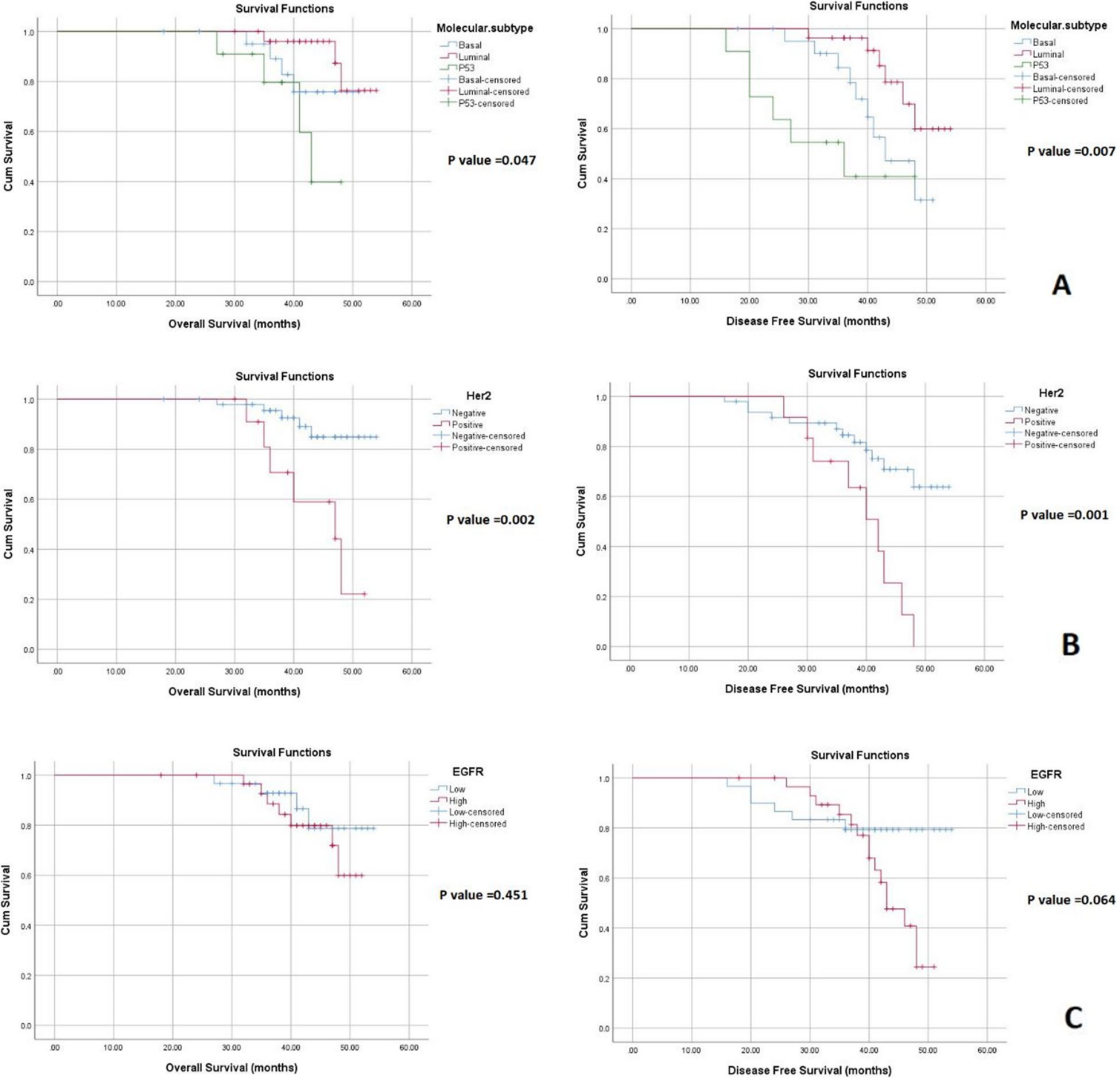
Kaplan-Meier survival curve showing. **A**: The relation between molecular subtypes of MIBC and DFS and OS. **B**: The relation between HER2 expression in MIBC and DFS and OS. **C**: The relation between EGFR expression in MIBC and DFS and OS

**Table 4:** Cox regression analysis of prognostic variables for overall survival and disease- free survival

Variables	OS		DFS	
	P value	OR (95% CI)	P value	OR (95% CI)
Molecular subtypes #	0.003		0.001	
Basal	0.006	0.048 (0.005–0.415)	0.005	0.104 (0.022–0.504)
Luminal	0.027	12.643 (1.325–120.587)	0.004	6.664 (1.839–24.154)
HER2 status	0.001	76.891 (5.937–995.835)	< 0.001	14.903 (3.302–67.260)
LV invasion	0.025	6.521 (1.271–33.460)	0.001	7.377 (2.178–24.987)

*P values less than 0.5 was considered statistically significant

# P53-WT is the reference subtype

### Relation between molecular subtypes of MIBC and response to treatment and fate

The basal subtype showed a significantly better response to NAC compared to luminal and p53-WT subtypes (*p* value = 0.045). Almost half of the basal subtype cases (10/22,45%) achieved CR Five cases (18.5%) of luminal subtype and only one case (9.1%) of p53-WT subtype achieved CR. No statically significant difference among the 3 subtypes regarding recurrence or death was found.

### HER2 and EGFR in molecular subtypes of MIBC

**Fig. 7:**
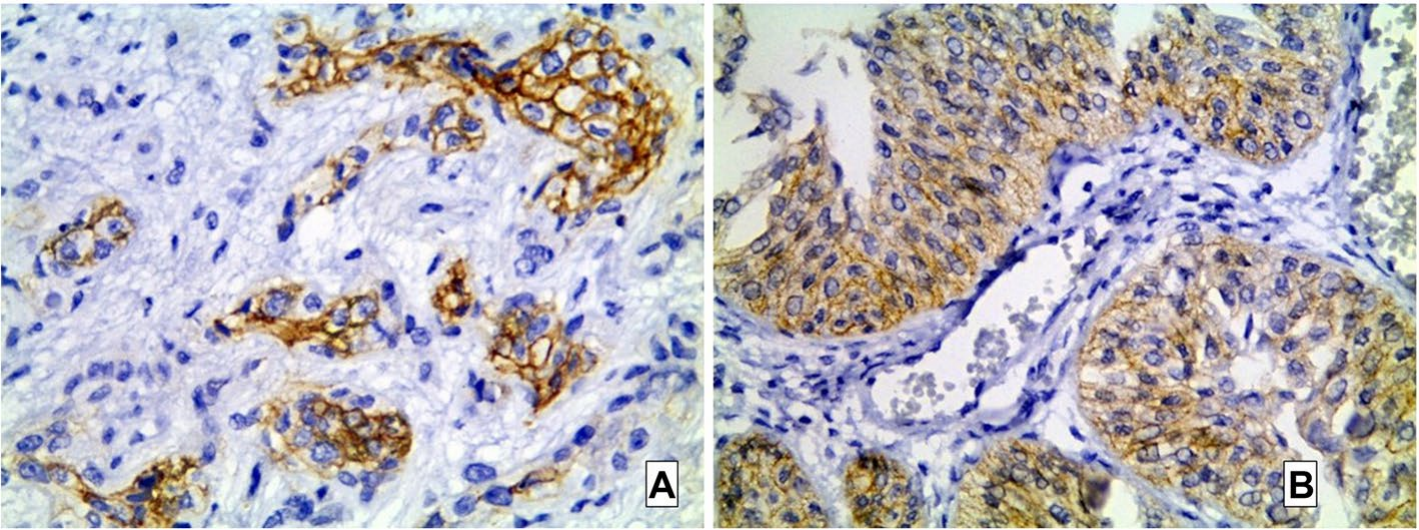
HER2 immunostain in studied MIBC cases. **A**: UC case showing + 3 HER2 positivity (× 400). **B**: Another UC case with + 2 HER2 (× 400)

HER2 was positive in 12 cases (20%). Eight cases were score 3+ and 6 cases were 2+ by IHC. Further FISH analysis of 2+ cases revealed HER2 amplification in 4 cases. HER2 expression was significantly distributed among the molecular subtypes of the studied cases. Most of HER2 positive cases 75% (9/12) were of luminal subtype. The remaining 3 HER2 positive cases were of basal subtype (25%) while none of the p53-WT cases showed HER2 positivity [Fig. 7]. EGFR gene expression was significantly different among the studied molecular subtypes. High EGFR expression was detected in all 22 cases (100%) of basal subtype, 6 cases (22%) of luminal and only 2 cases (18.2%) of p53-WT group. EGFR expression among molecular subtypes of MIBC was significantly different [*p* value < 0.001] with the basal subtype showing the highest EGFR expression.

### Relation between HER2 and EGFR expression and patient survival

Kaplan-Meier survival curves (Fig. 6B) analysis revealed a significant longer OS for HER2 negative (mean 51.85 months, 95%CI:49.59–53.57) than HER2 positive cases (mean 43.81 months, 95%CI:39.35–48.26) [*p* value = 0.002]. Regarding DFS, HER2 negative cases (mean 47.40 months, 95%CI:44.12–50.69) had a significantly longer survival than HER2 positive cases (mean 39.26 months, 95%CI:34.93–43.59) [*p* value =0.001]. Analysis of Kaplan Meier curves revealed no significant difference between EGFR in the studied cases and patient survival (OS and DFS) (Fig. 6C).

## Discussion

Molecular characterization of MIBCs has enabled their classification into subtypes that are associated with distinct clinic-pathological features. Bladder carcinoma has been categorized into two major subtypes; luminal and basal, that are similar to intrinsic types of breast carcinoma [2]. The utility of this modality in breast cancer resulted in more accurate prognostic and therapeutic patient stratification [22].

The basal subtype of bladder cancer express markers of primitive, basal urothelial cells such as high molecular weight keratins and has genetic similarities to basal breast tumors [1]. Luminal tumors were named due to their gene expression similarities to luminal breast tumors and positivity for markers of differentiated urothelium like GATA3, and the uroplakins. MDA group identified a subgroup of luminal tumors characterized by an activated p53 gene expression (p53-WT)(7).

A Meta analysis performed by Dadhania et al. [23] on large cohorts including 937 bladder cancer tissue samples showed that immunohistochemical expression of only two markers, one luminal specifically GATA3 and CK5/6 as basal are sufficient to classify bladder cancers into luminal and basal types with over 90% accuracy.

The aim of the present work was to determine the molecular subtype of the studied cases of MIBC using a panel of widely available immunostains [CK5/6,GATA3 and p53] following the Choi et al. stratification strategy [19]. Using the recommended cut-off of more than 20% tumor expression as positive, MIBCs included in the present study were stratified into distinct subtypes based on a nonoverlapping pattern of basal or luminal IHC markers.

Luminal tumors expressing GATA3 constituted most of our studied cases (27/60;45%) which was slightly lower than the MDA cohort and Lund group (70 and 82.5% respectively); the aforementioned studies had a total larger number of cases that included both invasive and noninvasive bladder tumors while the present study included only MIBCs. The tumor cancer genome atlas (TCGA) cohort included only muscle invasive high grade bladder tumors and reported a close percent of luminal subtype (52%). Subset of the luminal tumors expressing GATA3 were positive for activated p53(11/60,18.3%) and were assigned as p53-WT.

Basal subtype of MIBCs constituted 36.7% of our cases (22/60) which was slightly higher than basal MIBCs subgroup reported in MDA cohort and Lund (26 and 13.3% respectively) and close to the TCGA cohort who reported 44% basal tumors [23].

In the present study, the patients in the basal group were significantly younger than luminal and p53-WT groups (*p* value < 0.001). This age difference among subtypes coincided with other studies [24]. Most of the female patients included in this study (*n* = 10/16; 62.5%) were of basal subtype which is consistent with the findings of a prior pan-cancer molecular analysis that found MIBC to be a sex-biased cancer [25]. Same female predominance in basal tumors was detected by previous reports [26–28]. This sex specific subtype differences could be explained by the findings of Lin et al. [29] who previously discovered that androgen receptor mediated signaling promoted the growth of luminal tumors in MIBC.

Regarding the clinicopathological characteristics, tumor stage was significantly different among molecular subtypes. The basal group had a significant higher T4 cases (59.1%). This was consistent with previous studies [24, 30] that reported that basal tumors were enriched in patients with a more advanced clinical stage at diagnosis. Lymph node extension and LV invasion were significantly higher in basal group than luminal and p53-WT groups. This finding further supports that basal tumors are aggressive and have an increased risk of metastasis at presentation [31]. Histologically, all luminal tumors in this study showed conventional urothelial morphology while basal tumors showed squamous differentiation. This was explained by Warrick et al. who studied the associations between markers of molecular subtypes and histological variant in a retrospective cohort of 309 cystectomy specimens and concluded that squamous bladder cancer tends to be basal and that molecular subtype is related to the histological variant of bladder cancer [32].

OS and DFS were significantly longer in patients of luminal group than both basal than p53-WT groups included in the present study. This significant difference in patient outcome among molecular subtypes was consistent with other studies in which luminal tumors did significantly better than their basal tumor counterparts, with improved overall survival and disease-specific survival [33, 34]. Moreover, a meta-analysis by Parizi et al. confirmed that basal subtype is associated with adverse OS [35]. On the contrary, other studies reported a worse survival for the luminal group [36, 37]. The conflicting results could be attributed to different patient criteria and classification methods used in different studies. P53-WT subtype showed the worst DFS in the present study. This agrees with other studies that p53 wild signature had the worst survival following treatment. The intrinsic chemoresistance of p53 wild type renders these tumors nonresponsive to conventional NAC and hence the poor DFS survival for this group. Moreover, an important feature of p53-like tumors was the poor response to cisplatin based neoadjuvant chemotherapy [4].

The underlying mechanisms of drug resistance in MIBC are still being studied. MIBC is a highly heterogeneous disease in which one treatment for all cases was proved to be ineffective. Stratifying patients based on their molecular subtype can be utilized to predict their response to treatment. In the present study; patients in the basal group had significantly better response to treatment (*n* = 10/22;45%) than both p53-WT and luminal groups (*p* value = 0.045). This was in line with Razzaghdoust et al. [38] who studied the association of IHC detected MIBC subtype and NAC response and concluded that basal subtype was associated with CR. The results further confirms that patients with basal tumors identified through simple IHC have a higher possibility of achieving CR to NAC [30]. Basal tumors should be selected for NAC and not selected for radiotherapy given their hypoxic microenvironment [39].

In agreement with the preset study, multiple studies have concluded that NAC treatment response differs across the molecular subtypes of bladder carcinoma [1, 19, 36, 40, 41]. P53-WT group showed the highest number of non-responders 90% (n = 10/11) in the present work. This was in line with Fong et al. [40] that showed that the p53-like MIBC tumors were chemoresistant, while basal tumors were associated with the most survival benefit in patients treated with NAC. Finally, Choi et al. [19] observed that all of the p53-like MIBCs treated with NAC showed resistance. Moreover, p53- like signature was also associated with resistance to cisplatin-induced apoptosis. To establish the link between p53 subtype and chemoresistance in MIBC, gene expression profiles from a cohort of matched pre- and posttreatment tissue were compared. The lowest NAC response rate was seen in tumors initially classified as p53 subtype [42]. Furthermore, all drug resistant MIBC become p53 type after chemotherapy which implies an empirical role of p53 in drug resistance [4].

One study used gene expression profiles of MIBC patients treated with NAC to predict their treatment response and concluded that basal subtype was associated with better survival in the context of NAC and that p53-WT tumors were associated with chemoresistance and bone metastasis [36]. Molecular subtyping could help identify non-responders to NAC and spare them the ineffective time-consuming therapy [35]. Furthermore, Seiler et al. [41] demonstrated that the molecular stratification can predict patients response to NAC with basal tumors showing the highest response to preoperative cisplatin-based treatment regimen.

TCGA project established that bladder cancer has the third highest rate of mutation among all cancers, making it one of the top molecularly heterogeneous cancers [43]. Bladder cancer was proved to have differential enrichment of cell surface proteins, including EGFR and HER2, according to the molecular subtype [23].

Since EGFR and HER2 are predominantly expressed in the basal and luminal subtypes of bladder cancer, respectively, combining both targets provide a unique and highly selective way of targeting bladder cancers that have moderate expression of both [44]. The present study was extended to investigate HER2 expression and EGFR genetic alteration in relation to the molecular subtypes of MIBC.

In the present study, HER2 expression was found significantly different among the studied subtypes. In the present study, 75% of HER2 positive cases (9/12) were of luminal subtype and the remaining 3 HER2 + ve cases (25%) were of basal type. This was in agreement with Yorozu et al. [20] who reported that HER2 protein overexpression and gene amplification were specifically detected in the luminal subtype of urothelial carcinoma of renal pelvis and ureter (UCRPU), suggesting that these patients may benefit from HER2-targeted therapies like trastuzumab. Also Kiss et al. [45] studied HER2 alterations in MIBC and concluded that HER2 alterations were higher in the luminal subtype compared to basal. This finding indicates that this subset of MIBC cases which are HER2+ could benefit from directed antibody treatment. Basal subtype of MIBC is highly susceptible to chemotherapy, whereas the luminal subtype is considered chemo resistant [43]. Not all patients with HER2-positive cancers benefit from treatment with HER2-targeted therapies [46]. Therefore, adapting HER2-targeting therapy in the context of molecular subtypes could be a promising strategy mainly in luminal subtype.

In the studied cases of MIBC, DFS and OS were significantly shorter for HER2 positive than HER2 negative cases (*p* value 0.001 and 0.002 respectively). This finding was in agreement with Gan et al. [47] meta-analysis who concluded that high HER2 expression have a shorter recurrence-free survival and was related to poor prognosis. However, they found no effect on OS of patients. On the other hand Yorozou et al. [20] concluded in their study of UCRPU that the OS of patients with HER2 positive was significantly shorter than those with HER2 negative tumors. Because of limited number of patients in the present study which included a relatively high number of censored cases, the difference in survival among HER2 positive “luminal”, HER2 positive “basal”, HER2 negative “luminal” and HER2 negative “basal” subtypes of MIBC remains inconclusive. Future studies with large cohorts are required to further study the impact of HER2 changes on survival among different MIBC molecular subtypes.

EGFR signaling pathway was identified as a potential therapeutic target significantly dysregulated in bladder cancer especially basal type. Activation of EGFR pathway promotes multiple downstream signaling pathways which leads to increased cellular proliferation, invasion, and survival [48]. In the present work, EGFR was significantly higher in basal tumors than luminal and p53-WT subtypes (*p* < 0.001). This was in line with Eriksson et al. [10] who analyzed a cohort of 599 cases of urothelial carcinoma previously categorized to molecular subtypes for EGFR gene expression and genomic alterations and concluded that increased EGFR mRNA and protein levels were largely confined to the basal subtype [49]. EGFR is overexpressed in bladder cancer and associated with patient survival. However, in the present study, no significant association was found between EGFR and patient survival which could be due to small sample size.

In the basal subtype of MIBC, expression of EGFR and its ligands (heparin-binding EGF-like growth factor, and transforming growth factor alpha), and EGFR downstream targets (MYC, Interleukin-8 and SOX9) were significantly increased. Given these findings EGFR pathway could be considered as a key therapeutic target candidate for basal tumors. The basal subtype shows substantial molecular similarities with the basal-like subtype of breast cancer and the squamous cell carcinoma subtype of lung cancer. Accordingly, EGFR targeted therapies in these tumor types may be beneficial in treatment of MIBC basal subtype. Basal tumors have demonstrated sensitivity to EGFR inhibitors in preclinical data. However, epithelial mesenchymal transition (EMT) characteristic of basal tumors causes resistance to EGFR inhibitors in bladder carcinoma cells. Thus, epigenetic agents (e.g. histone deacetylase inhibitors) could be used to reverse EMT and promote sensitivity to EGFR inhibitors [36].

## Conclusions

MIBC can be stratified using a simple IHC panel [GATA3,CK5/6,P53] into clinically relevant molecular types that provide significant prognostic data and can predict patient response to treatment. Basal tumors are aggressive and respond well to chemotherapy while luminal tumors have better overall survival. P53-WT tumors are chemoresistant and require further treatments. HER2 and EGFR are potential therapeutic targets for MIBC in the context of molecular subtypes where luminal tumors are more likely to benefit from HER2 and basal tumors from EGFR directed therapies.
